# Preterm birth: pathogenesis and clinical consequences revisited

**DOI:** 10.1007/s00281-020-00809-w

**Published:** 2020-09-22

**Authors:** Anke Diemert, Petra Clara Arck

**Affiliations:** Department of Obstetrics and Fetal Medicine, University Medical Center Hamburg-Eppendorf, Hamburg, Germany

Preterm birth, defined as delivery at less than 37 weeks’ gestation, is one of the most pressing clinical problems in obstetrics worldwide [1]. Despite the significant medical advances accomplished over the last few decades, approximately 15 million babies are still born too soon, with alarming rates of rising prematurity in most countries [2, 3].

Preterm birth is not only the major contributor to neonatal morbidity and mortality globally, it also accounts for the increasing numbers of intergenerational non-communicable diseases. It is estimated that 106 million disability-adjusted life years are lost annually to preterm birth. Thus, preterm birth is a high priority public health problem and it is essential to reduce the rate of preterm birth in order to achieve the health-related Millennium Developmental goals set forth by the WHO in 2000 [4].

These goals can only be achieved if understanding of the pathogenesis of the multifactorial nature of preterm birth improves. Hence, a significant number of research endeavors aim to unearth markers and mediators accounting for the onset of preterm birth. In this special issue, we have compiled a comprehensive update on clinical, socioeconomic, and pathophysiological aspects currently known to be involved in preterm birth. Deindl and Diemert highlight features of the heterogeneity of the syndrome and focus on its connection with health care structures. The authors further summarize the structural modalities required for the prevention of preterm birth by discussing various care levels of hospitals [5].

To date, the accurate prediction and subsequently prevention of preterm birth is not yet within reach. Clearly, such prediction-based prevention of preterm birth is a highly desired goal and, thus, pursued by a number of researcher and clinicians. Pizzella et al. put forward evolving techniques to predict preterm birth, focusing on cervical imaging technologies. Currently used and emerging imaging techniques such as brightness-mode ultrasound, Raman spectroscopy, and photoacoustic endoscopy are discussed in the light of their advantages and disadvantages in assessing cervical remodeling. The authors propose that a combination of several imaging approaches, combined with clinical epidemiologic characteristics, holds the potential to accurately predict preterm birth [6]. Along this line, Petersen et al. discuss a timely concept which seeks understanding of the chronologically paced adaptations that synchronize the so-called pregnancy immune clock, taking advantage of multiomic approaches. Key elements which have the potential to program the immune clock of pregnancy are being proposed, including fetal, placental, and maternal pacemakers [7]. These evolving new techniques have not only strengthened the biological plausibility of immunological findings, they have also changed clinical practice in prenatal medicine.

Since dysfunctions of maternal immune adaptations have been linked to the onset of preterm birth, Green and Arck examine the current knowledge of the bidirectional communication between fetal and maternal systems and its role in the immunopathogenesis of preterm birth [8]. Much research in recent years has focused on using animal models to recapitulate the pathophysiology of preterm birth. Thus, the authors discuss the available evidence arising from mouse models as well as human studies, which supports that preterm birth results from a breakdown in fetal-maternal immune tolerance.

Menon et al. also acknowledge the importance of fetal-maternal immune tolerance and focus in their review article on inflammation of fetal membranes associated with preterm birth and preterm pre-labor rupture of the membranes. Both arise from distinct pathophysiologic pathways but share inflammation as a common underlying mechanism. Their review summarizes the mechanisms of fetal membrane cellular senescence, transitions, and the generation of inflammation that contributes to term and preterm parturitions [9].

Humberg and colleagues follow-up on these aspects and discuss sustained inflammation in the context of preterm birth and resulting consequences for the neonate. Here, the onset of chorioamnionitis is suggested to act as a first inflammatory hit. Postnatal confrontation of the prematurely born neonate with a drastically altered antigen exposure, such as hospital-specific microbes, artificial devices, drugs, nutritional antigens, and hypoxia or hyperoxia can then create a second inflammatory hit [10].

Blois and colleagues introduce galectins, an evolutionary conserved family of glycan-binding proteins, a pivotal mediator controlling fetal-maternal immune tolerance. In their review, the functional role of galectins in shaping cellular circuits that characterize a healthy pregnancy is comprehensively discussed. Moreover, the current understanding of galectins in term and preterm labor and the contribution of galectin-glycan circuits as key immunological pathways sustaining maternal tolerance and preventing microbial infections is proposed. This deeper understanding of the glycoimmune pathways regulating early events in preterm birth could provide a broad translational potential for the treatment of preterm birth [11].

Bayar et al. follow-up on microbial challenges, but also microbiome-mediated health advantages during pregnancy. In their review article, the complex interplay between the vaginal microbiome in pregnancy and its association with preterm birth is discussed in depth. Advances resulting from an improved understanding of the human microbiome are proposed, such as an improved awareness of the functional role of vaginal microbiota in both health and in disease [12].

Fetal medicine opens a new territory by treating the fetus as a patient. Hence, fetal therapy can improve perinatal survival or prevent severe long-term handicap. In utero fetal interventions can be divided into ultrasound-guided minimally invasive procedures, fetoscopic procedures, and open hysterotomy procedures, which carry an inherent risk of ruptured membranes and preterm birth. Therefore, Valenzuela et al. comprehensively discuss fetal therapies and their influence on preterm birth. They summarize the conditions that may benefit from fetal therapy and review the current therapies on offer in the light of their risk of ruptured membrane and preterm birth [13].

In summary, preterm birth has dire consequences for mother, child, and society in general. Babies who survive preterm birth often face disabilities, such as cerebral palsy, lung disease, blindness, and deafness. Continuous research endeavors addressing these not only persistent but also growing inequalities are urgently needed. With the present special issue addressing key features of the pathogenesis as well as clinical consequences of preterm birth, we sought to provide an updated platform summarizing current insights and future directions. This shall aid researcher and clinicians alike to continue striving for a deeper knowledge of preterm birth in order to lowering its rate globally.
